# Electronic health record (EHR) training program identifies a new tool to quantify the EHR time burden and improves providers’ perceived control over their workload in the EHR

**DOI:** 10.1093/jamiaopen/ooz003

**Published:** 2019-03-21

**Authors:** Yumi T DiAngi, Lindsay A Stevens, Bonnie Halpern – Felsher, Natalie M Pageler, Tzielan C Lee

**Affiliations:** 1 Department of Internal Medicine, Primary Care, Sutter Health/Palo Alto Medical Foundation, San Carlos, California, USA; 2 Department of Information Services, Stanford Children’s Health, Stanford, California, USA; 3 Department of Pediatrics, Stanford University School of Medicine, Stanford, California, USA

**Keywords:** EHR training, provider satisfaction, workload metrics

## Abstract

**Objective:**

To understand if providers who had additional electronic health record (EHR) training improved their satisfaction, decreased personal EHR-use time, and decreased turnaround time on tasks.

**Materials and Methods:**

This pre-post study with no controls evaluated the impact of a supplemental EHR training program on a group of academic and community practice clinicians that previously had go-live group EHR training and 20 months experience using this EHR on self-reported data, calculated EHR time, and vendor-reported metrics.

**Results:**

Providers self-reported significant improvements in their knowledge of efficiency tools in the EHR after training and doubled (significant) their preference list entries (mean pre = 38.1 [65.88], post = 63.5 [90.47], *P* < .01). Of the 7 EHR satisfaction variables, only 1 self-reported variable significantly improved after training: Control over my workload in the EHR (mean pre = 2.7 [0.96], post = 3.0 [1.04], *P* < .01). There was no significant decrease in their calculated EHR usage outside of clinic (mean pre = 0.39 [0.77] to post = 0.37 [0.48], *P* = .73). No significant difference was seen in turnaround time for patient calls (mean pre = 2.3 [2.06] days, post = 1.9 [1.76] days, *P* = .08) and results (mean before = 4.0 [2.79] days, after = 3.2 [2.33] days, *P* = .03).

**Discussion:**

Multiple sources of data provide a holistic view of the provider experience in the EHR. This study suggests that individualized EHR training can improve the knowledge of EHR tools and satisfaction with their perceived control of EHR workload, however this did not translate into less Clinician Logged-In Outside Clinic (CLOC) time, a calculated metric, nor quicker turnaround on in box tasks. CLOC time emerged as a potential less-costly surrogate metric for provider satisfaction in EHR work than surveying clinicians. Further study is required to understand the cost-benefit of various interventions to decrease CLOC time.

**Conclusions:**

This supplemental EHR training session, 20 months post go-live, where most participants elected to receive 2 or fewer sessions did significantly improve provider satisfaction with perceived control over their workload in the EHR, but it was not effective in decreasing EHR-use time outside of clinic. CLOC time, a calculated metric, could be a practical trackable surrogate for provider satisfaction (inverse correlation) with after-hours time spent in the EHR. Further study into interventions that decrease CLOC time and improve turnaround time to respond to inbox tasks are suggested next steps.

## BACKGROUND AND SIGNIFICANCE

Widespread adoption of the electronic health record (EHR) during the last decade may have led to advances in medical practice such as reductions in resource utilization, improvements in patient safety, and efficiency in care in some clinical areas.^1–5^ However, mitigating these positive findings, an Agency for Healthcare Research and Quality study concluded the impact on patient safety and quality of care are uncertain.^6^ In addition, growing dissatisfaction with the counterproductive byproducts of EHR in daily practice has led clinicians to speak out against the effects of EHR adoption on patient care, burnout, and professional fulfillment.^7–17^ The EHR, a tool intended to improve our ability to care for patients, has had the unintended consequence of impairing efficiency of practice, largely because of poor usability, the requirements for its use in regulatory reporting, and a shift of clerical data entry work to clinicians.^18^^,^^19^ The rising burden of these tasks for clinicians may well have increased the amount of time clinicians spend working in the EHR during personal time, decreasing an important component of professional satisfaction: work-life balance.^11^^,^^12^^,^^19^^,^^20^ In response, health systems are investigating ways to improve the provider experience, with a key priority being to enable provider efficiency within the EHR. An individual approach is to provide targeted EHR training to clinicians.

Previous training programs have evaluated EHR training at the time of the EHR implementation usually in the hospital setting.^21–23^ Other studies compared training approaches. A one-on-one training approach had the highest satisfaction and best perceived effectiveness in improving EHR efficiency over small groups, classrooms, and the e-learning approach.^24^ The outcomes of training interventions focused on use of EHR tools like order sets, templates, and management of medication and problem lists.^25^ One small study evaluated provider experience in the EHR as an outcome. This study of 57 experienced EHR users reported significant improvement in job satisfaction.^26^ For experienced EHR users, little is known about whether training improves efficiency using the EHR. Specifically, no studies have evaluated the impact of training on EHR time after clinic hours. To our knowledge, this intervention is the first to evaluate the impact on a calculated metric of EHR time after clinic hours.

## OBJECTIVE

The objective of this project was to measure changes before and after additional EHR training in the following areas: provider satisfaction measures related to the EHR and their workload, reported hours spent during personal time doing EHR-related work, calculated EHR time after clinic hours, and the turnaround time on patient messages and results.

## MATERIALS AND METHODS

### Program

The Home 4 Dinner program is an individualized provider training program based in adult learning theory designed to improve providers’ experience and efficiency with the EHR.^24^ Individualized learning plans were created for providers using 3 inputs: (1) a needs assessment survey completed by the providers before the training began; (2) vendor generated EHR-use measurements, including turnaround time on patient messages; and (3) an observation session using a standardized checklist, in which the provider was observed interacting with EHR during clinical care.^24^ Most providers subsequently participated in tailored one-on-one learning sessions with trainers intended to address the individual’s pain points. Providers in the ambulatory setting were given individual training sessions while inpatient providers received group training. Each training session lasted 1 to 2 h. Providers could sign up for a minimum of one session and as many sessions as they wished. The training team required 2 full time, nonclinician trainers, a training coordinator, and a part-time physician advisor.^27^

Over 700 primary care, obstetric, behavioral health, and subspecialty providers from inpatient and ambulatory settings were invited to participate. Academic faculty providers within certain departments were incentivized by an end-of-the-year performance bonus to participate in the presurvey, observation, and training. The postsurvey was optional for all participants.

### Participants

Stanford Children’s Health is an academic children’s health organization with an academic teaching hospital (303 beds) and affiliated ambulatory clinics for children and expectant mothers. The health system has 60 ambulatory practices across Northern California and approximately 500 000 ambulatory visits per year. In the spring of 2014, the health system moved from the inpatient-only use of the EHR Cerner (Kansas City, MO) to Epic Systems (Verona, WI) for both the inpatient and ambulatory settings. At the time of the Epic go-live starting May 2014, all clinicians received 5–10 h of group-based EHR training with additional training support tapered over 3 months. The Home 4 Dinner program was implemented starting January 2016, approximately 20 months post go-live as one approach to address burnout by improving the practice experience with the EHR.

### Measures

#### Self-reported clinician metrics

Both before- and after-training surveys were administered. The presurvey was a requirement to receive training and the department bonus. The presurvey contained a combination of questions related to perceived experience with the EHR and professional satisfaction, and questions about functionalities within the EHR (see Supplementary Appendix A). The providers were asked about 8 EHR/practice experience variables: satisfaction with the EHR, clinical work, face-to-face time with patients, workload in the EHR, satisfaction with the amount of time spent in the EHR after clinic hours, competence with the EHR, stress level related to the EHR, and the self-reported amount of time spent in the EHR after clinic (hours). These questions about perceived experience with the EHR were adapted from the Mini-Z burnout survey, now in use by the American Medical Association.^28^ Questions about EHR functionality included: 7 questions on frequency of use of various functions in the EHR, 7 questions about knowledge of common EHR functions, and 5 questions regarding the ease of using these EHR functions. The features and functions in the EHR we prioritized included: SmartPhrases, preference lists, filters, and closing visit notes. SmartPhrases are a type of shortcut tool that uses a keyboard “dot phrase” that when entered, inserts predefined data or text into the note. InBasket is equivalent to an inbox for clinical tasks, such as reviewing results, messages, and medication refills. We use the term “inbox” to refer to Epic’s InBasket. We also assessed ongoing use of dictation and use of the mobile version of the EHR.

The after-training survey was optional and included a shortened version of the same questions in the before-training survey, and questions about the training program. To increase the response rate, we also administered the shortened after-training survey in an email that the provider could easily respond to. Question responses in both surveys used a 5-point Likert scale (see Supplementary Appendix A).

#### Vendor-reported metrics

Starting in the spring of 2015, our EHR vendor provided a monthly report with metrics related to individual provider use of the EHR in our health system. These metrics included a range of raw data from the “average number of patients seen per day” to calculated metrics such as “review your patient calls messages quickly.” A standard set of metrics could be queried for each provider and we manually extracted the EHR-use metrics that were relevant for our study. We compared measures pre- and post-training by using data from the months of December 2015 and April 2017; the training started after December 2015. We excluded providers with data from less than 8 days of EHR use in either of those months which was the equivalent of about 50% of expected usage for a month (using a 4 day clinical work week).

#### Calculated EHR time outside of clinic metric—“CLOC”

A time-stamped log exists for EHR activity. We developed an algorithm that tracks the amount of time that a clinician is logged into the EHR after clinic hours (ie, during the evening, weekend, and vacations). We call this metric, CLOC Time or Clinician Logged-In Outside Clinic Time. Standard clinic hours are defined as a half hour before the start of the first scheduled appointment through the end time of the day’s last visit plus 1 h. Availability is the number of hours a provider is open to see patients. We calculated a ratio of hours using the EHR outside of scheduled patient care time to the number of hours a provider is open/scheduled to see patients. Note that our metric did not account for hours spent on inpatient service and thus would overestimate the ambulatory work after hours for providers with significant inpatient service time. The metric also did not account for other nonclinical work in the EHR (quality improvement or other administrative work, chart review for research, etc.) so could overestimate after-hours work for providers with significant nonclinical duties that required EHR access. With the exception of a few locations, as there was an inactivity time-out of 30 min, CLOC could be an overestimate of time spent doing EHR-related work. It is also possible to have an underestimate in CLOC time confounding this calculation in our academic setting. As many academic clinicians have administrative, educational, and research time built into their normal work day, this time gets used instead to complete EHR work, displacing the administrative work to after hours. This displaced EHR work that would normally occur after hours and now occurs within the hours of 8 am to 6 pm leads to an underestimate of CLOC. Calculations were done for each month from January 2015 to June 2017. The pretraining clinic hours were calculated as an average for the months of October through December 2015 and post-training hours were calculated as an average of April through June 2017. Providers who practiced only in the inpatient setting and clinicians with no availability in their schedule to see patients were excluded from analysis.

### Statistical methods

Descriptive statistics including frequencies, percentages, means, medians, and standard deviations were calculated for all variables. Paired *t*-tests were used to compare differences between before and after responses for each question and each numeric value for the EHR-use metrics. Analysis of variance was used to compare differences within groups for a variable. Less than 5% of data were missing for any variable; and thus, we kept these data as missing rather than impute or average data to fill-in. We analyzed data using JMP Pro version 12.1.0 (SAS Inc., Cary, NC). Statistical tests were 2-tailed, using an alpha of 0.01. Because this study was exploratory in nature, we performed multiple comparisons. We did not do a formal statistical correction for multiple comparisons, but used a slightly stricter *P*-value (<.01) to decrease the likelihood of a type I error. The study was exempted from review by the Stanford University Institutional Review Board.

## RESULTS

### Demographics/practice characteristics

Our total response rate was 26%, which is similar to other studies.^29^^,^^30^ Of the 561 eligible participants, 147 (26%) completed the entire training program with both the before- and after-surveys for inclusion in the study analysis with additional exclusions in parts of the analysis for missing data from vendor-reported metrics or CLOC metrics as stated above. There was no significant difference between those who did and did not complete the after-training survey with respect to gender, years in clinical practice, years using an EHR, and clinical specialty (*P* > .05). Among the individuals who completed the before- and after-training surveys, most were physicians (92%) and were in practice for more than 5 years after training (74%). The participants were also experienced EHR users, with a majority having more than 3 years of prior EHR experience (84%). The 147 providers who completed the study practiced primarily in an academic or ambulatory setting (86%, 85%, respectively) with 72% of them practicing at least more than 1 day per week (3 half day clinical sessions or more per week). See Table 1 for more details.

**Table 1.** Participant demographics and practice characteristics

	Completed entire training program	Eligible for training program
	(*N* = 147)	(*N* = 561)
Gender		
Female	94 (64.0)	389 (69.3)
Practice setting		
Ambulatory	125 (85.0)	505 (90.0)
Inpatient	22 (15.0)	56 (10.0)
Practice setting 2		
Academic	127 (86.4)	492 (87.7)
Community	20 (13.6)	69 (12.3)
Specialty^a^		
Primary care/adolescent	21 (14.3)	86 (15.3)
Pediatric subspecialty	93 (63.3)	371 (66.1)^b^
Obstetrics and gynecology (adult medicine)	3 (2.0)	
Surgical subspecialty	16 (10.9)	58 (10.3)
Behavioral health	14 (9.5)	46 (8.2)
Years in clinical practice after all training (years)		
1–2	14 (9.5)	68 (12.1)
3–5	25 (17.0)	96 (17.1)
6–10	32 (21.8)	100 (17.8)
11–15	17 (11.6)	88 (15.7)
16–20	13 (8.8)	66 (11.8)
20+	46 (31.3)	143 (25.5)
Years using an EHR (years)		
<1	2 (1.4)	13 (2.3)
1–2	22 (15.0)	90 (16.0)
3–5	38 (25.9)	144 (25.7)
5+	85 (57.8)	314 (56.0)
Half day clinical sessions per week (*N*=92)		
1–2 (10% time)	26 (28.3)	
3–6 (part time)	43 (46.7)	
≥7 (full time)	23 (25.0)	

*Abbreviation:* EHR, electronic health record.

a Specialties: Pediatric subspecialty includes anesthesia, pain, neonatology, pediatric hospitalist; Surgical subspecialty includes inpatient anesthesia, Otorhinolaryngology, ophthalmology; see Supplementary Appendix B for the full list of specialty breakdown.

b Represents Pediatric Subspecialty plus Obstetrics and Gynecology combined.

### Before-training data

#### EHR usage patterns

Prior to training, 15% reported using no personal smart phrases and 71% reported using 5 or fewer smart phrases (Table 2). A majority of providers (69%) responded that it was “very important” or “extremely important” to complete their office notes the same day; yet only 58% of participants reported being able to “most of the time” or “always” complete their EHR notes the same day. Only 18% of participants reported reviewing and responding to inbox messages the same day and 31% reported taking a week or longer to review and respond.

**Table 2.** Participant reported EHR usage patterns before training among those who completed both the before- and after-training survey

	Clinicians
	(*N* = 147)
Personal smart phrase use on a regular basis	
0	22 (15.0)
1–5	82 (55.8)
6–9	8 (5.4)
10 or more	35 (23.8)
Importance of completing notes on the same day (*N*=134)	
Not at all important	3 (2.2)
Unimportant	11 (8.2)
Neither important nor unimportant	28 (20.9)
Very important	53 (39.6)
Extremely important	39 (29.1)
Complete notes (close encounters) on the same day (*N*=134)	
Never	2 (1.5)
Rarely	26 (19.4)
Sometimes	29 (21.6)
Most of the time	55 (41.0)
Always	22 (16.4)
Time to review and respond to inbox messages	
I don’t use	5 (3.4)
Longer than a week	9 (6.1)
Within the same week	37 (25.2)
Within 1–2 days	69 (46.9)
Within the same day	27 (18.4)

#### Correlation between self-reported and CLOC time in the EHR after hours

**Figure 1.**
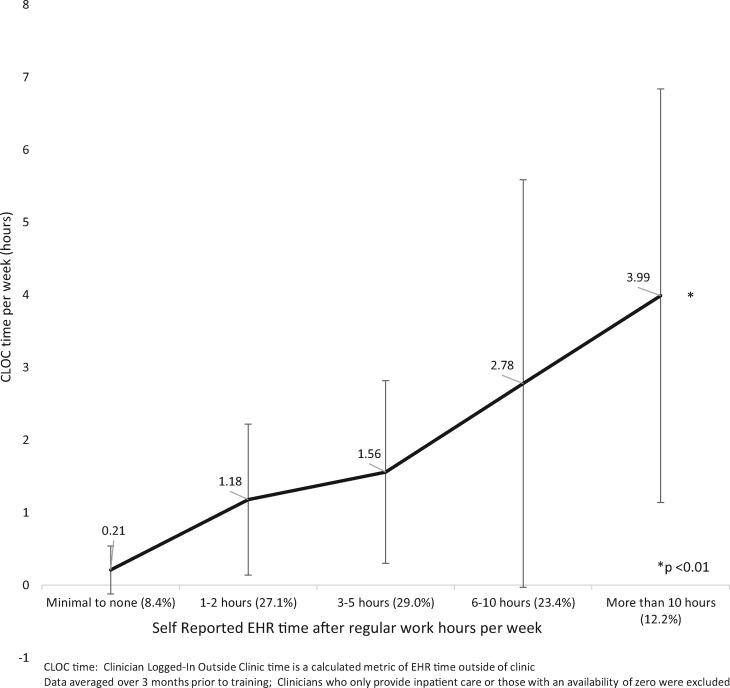
Relationship between self-reported and calculated CLOC time before training. CLOC, Clinician Logged-In Outside Clinic.

Self-reported time and CLOC time were correlated (Pearson’s *R* = 0.9864, *P* < .01) that is the more time a provider reports spending after clinic in the EHR, the more time we calculated from time-log data (Figure 1). However, the ratio of self-reported versus measured CLOC time is 2–3 to 1 not 1–1.

#### Correlations between EHR experience and EHR after-hours usage

**Figure 2.**
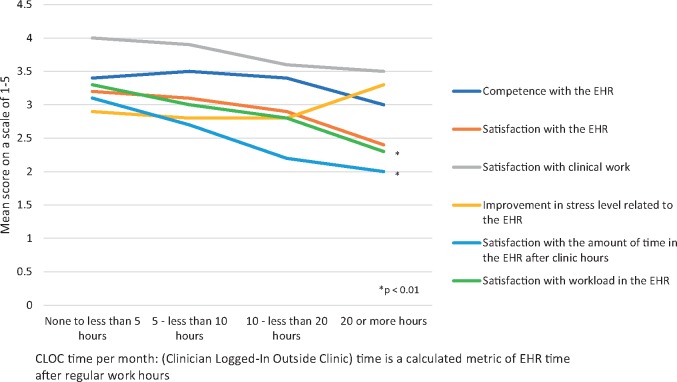
Relationship between CLOC metric and self-reported EHR experience. CLOC, Clinician Logged-In Outside Clinic; EHR, electronic health record.

We compared the impact of the amount of EHR usage after clinic hours with the following EHR experience variables: Satisfaction with Epic, work stress level, satisfaction with the amount of time spent in the EHR after clinic hours, control over workload, satisfaction with clinical work, and self-rated competence with the her (Figure 2). Figure 2 compares the calculated CLOC time metric to their corresponding EHR experience variables. Significant differences were seen in both self-reported EHR hours and CLOC time for the 2 variables: satisfaction with workload (mean range 2.0–3.4, 2.3–3.3, respectively, *P* < .01) and satisfaction with the amount of time spent in the EHR after clinic hours (mean range 1.7–4.0, 2.0–3.1, respectively, *P* < .01).

### After-training program feedback

Forty-four percent had 1 training session, 40% had 2 sessions, and 16% had 3–5 sessions. Regarding feedback for the training program, on average, participants found the training session helpful to improve their use of the EHR (3.8 of 5, SD = 1.07, *N* = 94). The question is: “Was the training session(s) helpful to improve your use of Epic?” (Very helpful, somewhat helpful, neutral, not very helpful, not at all helpful). There was no significant difference in those reporting the training sessions helpful to improve their use of the EHR by years of practice (more than 20 years: [mean 4.2 (0.58), *P* = .01] vs less than 20 years: [mean range 2.9–3.9]) nor by the amount of self-reported time in the EHR after clinic hours (more than 10 h per week: [mean 4.08 (0.51), *P* = .05] vs less than 10 h per week: [mean range 3.3–4.1]). In response to the questions about enrolling in additional training and recommending others to training, responses had a mean of 3.0 (SD = 1.16, *N* = 94) and 3.5 out of 5 (SD = 1.05, *N* = 147), respectively. We conducted an analysis of variance and found no significant difference in the program feedback by specialty, gender, number of training sessions, years of previous EHR use, nor by self-reported EHR competence prior to training.

### Comparison of change after training

#### EHR experience variables

We observed that EHR experience improves following the Home 4 Dinner training in the following experience variable: Perceived workload within the EHR (mean pre = 2.7 [0.96], post = 3.0 [1.04], *P* < .01; Table 3). Self-reported hours spent in the EHR after clinic (mean pre = 5.0 h [4.28], post = 4.1 h [3.68], *P* = .02) following training did not significantly improve. No significant difference was observed in the satisfaction variables of EHR and clinical work.

**Table 3.** EHR experience variables before and after training

	Mean (SD)	Mean (SD)
Survey questions	Before training	After training
Satisfaction with		
The EHR (*N* = 147)^	3.0 (1.0)	3.0 (1.0)
Clinical work (*N* = 147)^	3.9 (0.8)	3.8 (0.8)
Control over workload in the EHR (*N* = 114)^	2.7 (1.0)	3.0 (1.0)*
Amount of time in the EHR after clinic hours (*N* = 94)^	2.7 (1.1)	2.7 (1.0)
Competence with the EHR (*N* = 147)^	3.3 (0.9)	3.4 (0.9)
Improvement in stress level related to the EHR (*N* = 94)^a^	2.7 (0.9)	2.9 (0.8)
Self-reported hours spent in the EHR after clinic per week (in hours) (*N* = 94)	5.0 (4.3)	4.1 (3.7)

a Scale of 1–5 where a higher number indicates a more favorable rating.

* *P* < .01.

#### EHR functionality variables

**Figure 3.**
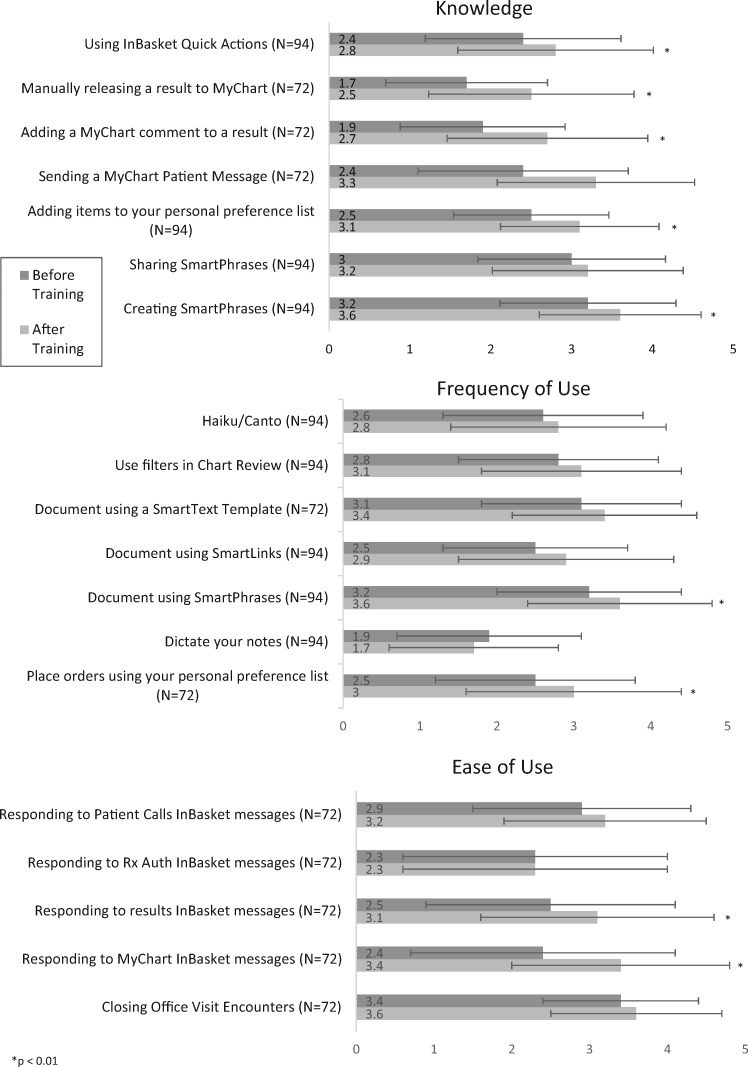
EHR functionality (self-reported). EHR, electronic health record.

We saw significant differences before and after training across multiple EHR functionality variables of knowledge, EHR tool use, and ease of tool use (Figure 3). Across all knowledge variables compared with before training, after training participants’ scores improved in all 7 items including “Creating SmartPhrases,” “Adding a MyChart comment to a result,” and “Using InBasket Quick Actions.” Across frequency-of-use variables, participants’ scores significantly increased in 2 of 7 items including: “Placing orders using personal preference list” and “Document using SmartPhrases” after training. Finally, across ease-of-use variables, these 2 outcomes significantly increased after training: “Responding to MyChart InBasket messages” and “Responding to results InBasket messages.” We used a 5-point Likert Scale from not at all knowledgeable to extremely knowledgeable; never to always; very difficult to very easy, respectively.

#### Vendor-reported metrics—Provider response time variables

The number of preference list entries significantly increased by almost double (mean pre = 38.1 [65.88], post = 63.5 [90.47], *P* < .01) following training (Table 4). There was no significant difference in response time for inbox results (mean before = 4.0 [2.79] days, after = 3.2 [2.33] days, *P* = .03) nor response time to patient calls following training (mean pre = 2.3 [2.06] days, post = 1.9 [1.76] days, *P* = .08). No significant change was observed in completing office visits the same day of the appointment nor reviewing patient call messages quickly and notably, this is occurring at very low rates (0.5% of the time).

**Table 4.** Vendor defined EHR-use variables before- and after-EHR training

	Mean (SD)		Mean (SD)		*t*-test
EHR vendor metrics	Before training	Range	After training	Range	
Provider Response Time (%) (*N* = 91)					
Close office visits the same day (%)^a^	0.5 (0.4)		0.5 (0.4)		1.78
Reviews patient call messages quickly (%)^b^	0.5 (0.4)		0.6 (0.8)		1.18
Inbox turnaround time (days) (*N* = 65)					
Results	4.0 (2.8)	(0.04–11.5 days)	3.2 (2.3)	(0.1–12 days)	2.27
Patient calls	2.3 (2.1)	(0.1–10 days)	1.9 (1.8)	(0.1–7.7 days)	1.79
Preference list entries (*N* = 91)^c^	38.1 (65.9)	(0–256 entries)	63.5 (90.5)	(0–404 entries)	**5.06** *

*Notes:* One month of data before training and after training; Providers with less than 8 days of EHR use in either of those months were excluded.

a Definition: For each provider what percentage of days in the measurement period where at least 95% of their visits closed (completed) the same day.

b Definition: For each provider what percentage of days in the measurement period were at least 85% of their patient calls in your InBasket completed within 24 h.

c Definition: The number of items on the selected providers’ user preference lists.

* *P* < .01.

#### Calculated after-work time metric

**Figure 4.**
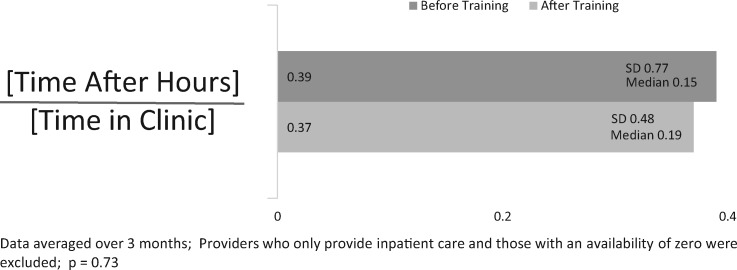
Mean calculated CLOC time in the EHR before and after training (*N* = 107). CLOC, Clinician Logged-In Outside Clinic; EHR, electronic health record.

This study showed no difference in our calculated metric for after-work hours (mean pre = 0.39 [0.77] to post = 0.37 [0.48], *P* = .73; Figure 4).

## DISCUSSION

Our program aimed to improve provider EHR work satisfaction, decrease personal EHR-use time, and decrease turnaround time on tasks by providing individually tailored, on-site EHR training with a nonclinician trainer. The program was successful in improving provider’s satisfaction with their perceived control over their EHR workload, however, the training program did not significantly decrease either the self-reported or the calculated time individuals are using the EHR during personal time. It seems counterintuitive that providers’ satisfaction increased post-training with their perceived “control over their EHR workload” although their actual workload appears to not have decreased. One explanation is that the content of our nature of the interactions during the training did increase physicians’ confidence in using the EHR tool and their perceived confidence over the work they do in the EHR. Other studies have shown that physicians reporting greater degrees of control over their clinical work were more likely to report high overall professional satisfaction.^19^

Although providers self-reported significant improvement in knowledge, usage, and ease of use of various EHR functions and tools after training, this also did not translate either into significant improvements in patient-facing tasks like response time to patient messages or results.

Our study was the first study to evaluate the impact of training on time-log data from the EHR. While there was not a significant impact on that metric, there were important correlations between that metric and provider experience which could be useful for future programs. The study provides support for previous work showing that providers spend significant hours doing clinical work in the EHR and an increasing percentage of this work occurs after clinic hours.^12^^,^^20^^,^^28^^,^^31^ Our study also showed correlations between EHR experience and measured EHR time, which is in line with previous work demonstrating that providers spending more than 6 h per week in the EHR after clinic hours were 3 times more likely to report burnout.^7^

### Limitations

This intervention is an initial, descriptive, exploratory operational study with multiple comparisons. Our intervention could have been more intensive as the majority of participants elected to participate in only 1–2 training sessions. The intervention only involved training the end user without systematic customizations for the department of common pain points. While we used a slightly stricter *P*-value to reduce the risk of type 1 error with multiple comparisons, further studies are warranted to confirm these results. Additional limitations of our study include the low completion rate which may in part be explained by lack of a financial incentive for the after-survey completion. Also there was nonrandom enrollment into the program—participation with the before-survey and training (after-survey completion was optional) was incentivized leading to response bias from more financially engaged providers. Additionally, our results represent the experience of a single, academic pediatric institution with a small community practice cohort of providers in full time clinical practice and very small adult medicine cohort. Finally, although our study compares changes between matched pairs, we did not have a control group and some of our improvements could be attributed to self-improved use of the EHR over time regardless of the training.

Despite these limitations, this is the first study to evaluate the impact of training on a calculated metric of EHR usage time. A notable challenge in EHR training and intervention programs are the costly resources involved in surveying clinicians. A surrogate metric for provider satisfaction, like CLOC time, would be a valuable tool for evaluating future interventions. We demonstrated that the CLOC metric correlated to provider’s self-reported EHR time. Additionally, the CLOC metric had a significant inverse correlation to satisfaction with perceived control over EHR workload and satisfaction with the amount of EHR use during personal time in preintervention data. For example, providers who were calculated to spend the least time in the EHR after clinic hours had the highest satisfaction with control over EHR workload and highest satisfaction with the amount of EHR use during personal time as would be expected.

In our clinician population, the majority of academic providers had some inpatient service time and research, teaching, and administrative time during the 3-month measurement period (75% in part time clinical practice, Table 1). The CLOC algorithm described in the “Materials and Methods” section is largely designed for an ambulatory population; it is not clear the ways inpatient-related EHR charting and EHR use during administrative and research time altered CLOC time. It would be worthwhile repeating this study using the CLOC metric in a purely outpatient, nonacademic practice setting with the majority of the providers in full time clinical practice. CLOC may need to be normalized to patient volume which we did not do in our study. A recent study validated EHR timestamp data to predict outpatient ophthalmology clinic workflow timings showing accuracy of the algorithm within 3 min of the observed time in more than 80% of the appointments.^32^ Finally, it is notable that there is 3-fold ratio of providers’ self-reporting of after clinic hours to what is actually measured to occur by our CLOC algorithm. Either providers are over-reporting their time, or our algorithm is under-measuring providers’ after-hour time in the system. This will require further study.

Future studies should also evaluate whether training can significantly impact efficiency in complex workflows, like completing a clinic note with associated billing and regulatory requirements (closing encounters)^33^ or whether real-time scribe support is more effective. Future studies should also evaluate how much training on EHR tool usage can significantly impact efficiency in the inbox work, as primary care clinicians are now spending up to 50% of their time on these tasks (responding to patient messages, prescription refills, and results review).^12^^,^^20^^,^^27^^,^^31^^,^^34^ For example, in our study, a significant increase in self-reported EHR tool use like smart phrases (a process), did not lead to significant improvements in turnaround time on tasks like results review or in response time to patient messages (outcomes).

The addition of this “virtual” clinical work to the unchanged responsibilities of in-person clinical work has largely led to measurable increases in personal time in the EHR and correlation to provider burnout.^7^ Future studies could evaluate the types of innovations, including workflow redesign aligned with new payment models that lead to decreased time frontline providers spend in the EHR after hours. Finally, it might be possible to improve the metrics we proposed at the expense of declining patient satisfaction (if physicians spend more time typing notes during visits), and poor quality and inaccurate notes (increased use of copy-forward without editing for accuracy) which would be an unintended consequence that should be included in future evaluation.

## CONCLUSION

Our pre-post study showed that in-clinic EHR training with a nonclinician can improve self-reported knowledge of EHR tools. It can improve satisfaction with perceived control of workload in the EHR even though it did not improve the amount of time clinicians are spending in the EHR during personal time, nor improve turnaround time on patient messages and results. This study showed that 1–2 sessions of in-clinic EHR training alone does not significantly improve efficiency in the EHR. CLOC time, a calculated metric of provider time in the EHR after clinic hours significantly correlated to metrics related to provider satisfaction. It may be a reliable tool for tracking ambulatory providers’ time and is a less-costly surrogate metric for provider satisfaction in EHR work than surveying clinicians.

## FUNDING

This research received no grant from any funding agency in the public, commercial, or not-for-profit sector.

## AUTHOR CONTRIBUTORS

All authors attested that they have made substantial contributions.

## SUPPLEMENTARY MATERIAL

Supplementary material is available at *Journal of the American Medical Informatics Association* online.

## Supplementary Material

Supplement_Material_ooz003
