# Supplementary material for: Electronic health record (EHR) training program identifies a new tool to quantify the EHR time burden and improves providers’ perceived control over their workload in the EHR
Source: JAMIA Open. 2019 Mar 21;2(2):222–30. doi: 10.1093/jamiaopen/ooz003 (PMC6952029; doi:10.1093/jamiaopen/ooz003)
Supplement: Supplement_Material_ooz003 [file supplement_material_ooz003.zip › Appendix A - SCHHome4DinnerSurvey.pdf]

Appendix A: Home for Dinner Needs Assessment Survey

This is an electronic survey that can be tracked and allows the user to comment as needed.

This survey is intended to determine areas where we can help and should take less than 15 minutes to complete. This is the first step in a program designed to help streamline your Epic usage. Please respond to each item candidly and provide comments to help us understand your responses. Individual responses to this survey will only be used for the purpose of creating your personal EHR training plan. Thank you for your time and input.

Complete below: The preferred contact information provided will be used to schedule your Home for Dinner program sessions.

Name:
Stanford Children's Epic Login:
Best Contact Phone Number:
Preferred Email:
Division:

How many years have you been in clinical practice after training?

❍ 1-2 years (1)
❍ 3-5 years (2)
❍ 5-10 years (3)
❍ 10-15 years (4)
❍ 15-20 years (5)
❍ 20+ years (6)

How many of those years have you been using ANY Electronic Health Record (EHR)?

❍ (1)
❍ 1-2 years (2)
❍ 3-5 years (3)
❍ 5+ years (4)

1. What is your overall level of competence with Stanford Children's Epic?

	Excellent (5)	Very Good (4)	Good (3)	Fair (2)	Poor (1)
Select	❍	❍	❍	❍	❍

2. My overall satisfaction with Epic is:

	Excellent (5)	Very Good (4)	Good (3)	Fair (2)	Poor (1)
	❍	❍	❍	❍	❍

3. My overall satisfaction with my clinical work is:

	Excellent (5)	Very Good (4)	Good (3)	Fair (2)	Poor (1)
	❍	❍	❍	❍	❍

4. My work stress level since the Epic implementation has:

	Significantly Improved (5)	Mildly Improved (4)	Not Changed (3)	Mildly Worsened (2)	Significantly Worsened (1)
	❍	❍	❍	❍	❍

Answer If 4. My work stress level since the Epic implementation has: - Significantly Worsened Is Selected Or 4. My work stress level since the Epic implementation has: - Mildly Worsened Is Selected

The top 3 things about Stanford Children's Epic that cause me stress are:

1.
2.
3.

5. The amount of time I spend in Epic outside routine work hours (i.e. 8am – 6pm Monday through Friday for a full-time clinician) is:

	None (5)	Minimal (4)	Satisfactory (3)	Moderately High (2)	Excessive (1)
(1)	❍	❍	❍	❍	❍

6. My control over my workload in Epic is:

	Optimal (5)	Good (4)	Satisfactory (3)	Marginal (2)	Poor (1)
(1)	❍	❍	❍	❍	❍

Comments on any of the above questions (optional):

7. How knowledgeable are you on the following Stanford Children's Epic functionality?

	Extremely Knowledgeable (5)	Knowledgeable (4)	Somewhat Knowledgeable (3)	Not Very Knowledgeable (2)	Not at all Knowledgeable (1)
Creating SmartPhrases (1)	❍	❍	❍	❍	❍
Sharing SmartPhrases (2)	❍	❍	❍	❍	❍
Using Keyboard Shortcuts (e.g. Alt+A) (20)	❍	❍	❍	❍	❍
Adding items to your personal Preference List (3)	❍	❍	❍	❍	❍
Organizing Preference List items (4)	❍	❍	❍	❍	❍
Placing Orders (5)	❍	❍	❍	❍	❍
Reviewing/Canceling Open Orders (6)	❍	❍	❍	❍	❍
Sending a MyChart Patient Message (7)	❍	❍	❍	❍	❍
Adding a MyChart comment to a result (8)	❍	❍	❍	❍	❍
Manually releasing a result to MyChart (9)	❍	❍	❍	❍	❍
Creating a Result Note (10)	❍	❍	❍	❍	❍
Using In Basket Quick Actions (11)	❍	❍	❍	❍	❍
Granting access to your In Basket (12)	❍	❍	❍	❍	❍
Attaching to another's In Basket (13)	❍	❍	❍	❍	❍
Editing your Dictionary (14)	❍	❍	❍	❍	❍
Using Spell Check when documenting a note (15)	❍	❍	❍	❍	❍
Using Auto-Correct to expand abbreviations and enhance typing efficiency (16)	❍	❍	❍	❍	❍
Accessing WebLinks (17)	❍	❍	❍	❍	❍
Postponing an In Basket message (18)	❍	❍	❍	❍	❍
Communicating visit documentation with other providers (19)	❍	❍	❍	❍	❍

Comments on your knowledge of Epic tools (optional):

8. How frequently do you use each of the following functions?

	Always (5)	Most of the Time (4)	Sometimes (3)	Rarely (2)	Never (1)
Place orders using your personal Preference List (1)	❍	❍	❍	❍	❍
Dictate your note (2)	❍	❍	❍	❍	❍
Document using SmartPhrases (3)	❍	❍	❍	❍	❍
Document using SmartLinks (e.g. .LASTLAB) (4)	❍	❍	❍	❍	❍
Document using a SmartText Template (5)	❍	❍	❍	❍	❍
Pin your note to the Sidebar (6)	❍	❍	❍	❍	❍
Use filters in Chart Review (7)	❍	❍	❍	❍	❍
Media Tab to find scanned documents (8)	❍	❍	❍	❍	❍
Haiku/Canto (9)	❍	❍	❍	❍	❍

Comments on use frequency of Epic tools (optional):

9. Rate the level of ease for using each of the following Stanford Children's Epic functions.

	Very Easy (5)	Easy (4)	Neutral (3)	Difficult (2)	Very Difficult (1)	N/A (0)
Documenting a routine Office Visit (1)	❍	❍	❍	❍	❍	❍
Finding and Placing Orders (2)	❍	❍	❍	❍	❍	❍
Closing Office Visit Encounters (3)	❍	❍	❍	❍	❍	❍
Sending In Basket messages (4)	❍	❍	❍	❍	❍	❍
Responding to MyChart In Basket messages (5)	❍	❍	❍	❍	❍	❍
Responding to Results In Basket messages (6)	❍	❍	❍	❍	❍	❍
Responding to Rx Auth In Basket messages (7)	❍	❍	❍	❍	❍	❍
Responding to Patient Calls In Basket messages (8)	❍	❍	❍	❍	❍	❍
Co-Signing Notes (9)	❍	❍	❍	❍	❍	❍
Co-Signing Transcriptions (10)	❍	❍	❍	❍	❍	❍

Comments on ease of use of Epic functions (optional):

10. How many personal SmartPhrases do you use on a regular basis?

❍ 0 (1)
❍ 1 (2)
❍ 2 (3)
❍ 3 (4)
❍ 4 (5)
❍ 5 (6)
❍ 6 (7)
❍ 7 (8)
❍ 8 (9)
❍ 9 (10)
❍ 10+ (11)

11. How often do you close your encounters on the same day?

	Always (5)	Most of the Time (4)	Sometimes (3)	Rarely (2)	Never (1)	N/A (42)
(1)	❍	❍	❍	❍	❍	❍

How important is it to you close your encounters on the same day?

	Extremely Important (5)	Very Important (4)	Neither Important nor Unimportant (3)	Unimportant (2)	Not at all Important (1)	N/A (36)
(1)	❍	❍	❍	❍	❍	❍

Identify any barriers to closing your encounters on the same day (optional):

12. How quickly do you review and respond to InBasket messages?

- ❍ Within the same day (4)
- ❍ Within 1-2 days (3)
- ❍ Within the same week (2)
- ❍ Longer than a week (1)
- ❍ I don't use InBasket (0)

Answer If 8. How quickly are you able to review and respond to InBasket messages? Within the same day Is Selected

If able to review/respond the same day, within how many hours of receipt do you usually respond?

- ❍ (11)
- ❍ 1 (10)
- ❍ 2 (9)
- ❍ 3 (8)
- ❍ 4 (7)
- ❍ 5 (6)
- ❍ 6 (5)
- ❍ 7 (4)
- ❍ 8 (3)
- ❍ 9 (2)
- ❍ 10+ (1)

Identify any barriers that keep you from responding to messages quickly (optional):

13. How many hours outside of your regular working hours (i.e. 8am – 6pm Monday through Friday for a full-time clinician) do you spend charting per week?

- ❍ (16) ____________________
- ❍ 1 (15)
- ❍ 2 (14)
- ❍ 3 (13)
- ❍ 4 (12)
- ❍ 5 (11)
- ❍ 6 (10)
- ❍ 7 (9)
- ❍ 8 (8)
- ❍ 9 (7)
- ❍ 10 (6)
- ❍ 11 (5)
- ❍ 12 (4)
- ❍ 13 (3)
- ❍ 14 (2)
- ❍ 15+ (1)

If >15 hours spent, please quantify:

Hours (1)

14. The quality of time I spend with patients during their face-to-face visit is:

	Excellent (5)	Very Good (4)	Good (3)	Fair (2)	Poor (1)
(1)	❍	❍	❍	❍	❍

15. The top 3 EHR functions that I would like help with are:

1. (1)
2. (2)
3. (3)

16. The top 3 things that I am the most satisfied with in Stanford Children's Epic are: (Optional)

1. (1)
2. (2)
3. (3)

17. My 3 wishlist items that I would like in Stanford Children's Epic are: (Optional)

1. (1)
2. (2)
3. (3)

Any additional comments?
