# Supplementary material for: Electronic health record (EHR) training program identifies a new tool to quantify the EHR time burden and improves providers’ perceived control over their workload in the EHR
Source: JAMIA Open. 2019 Mar 21;2(2):222–30. doi: 10.1093/jamiaopen/ooz003 (PMC6952029; doi:10.1093/jamiaopen/ooz003)
Supplement: Supplement_Material_ooz003 [file supplement_material_ooz003.zip › Appendix B Specialty Breakdown 2018.pdf]

Appendix B

Specialty Breakdown

Primary Care/Adolescent: General pediatrics, Adolescent medicine

Pediatric subspecialty: Anesthesia, Allergy and immunology, Cardiology, Rheumatology, Neurology, Endocrine, Genetics, Gastroenterology, Hematology and oncology, Infectious Disease, Neonatology and developmental medicine, nephrology, Pain management (outpatient anesthesia), Pediatric hospitalist, Pulmonology, PICU

Obstetrics and Gynecology (adult medicine): Obstetrics and gynecology, Maternal fetal medicine

Surgical subspecialty: Inpatient anesthesia, Otorhinolaryngology, Orthopaedics, Ophthalmology, Plastic Surgery, Urology

Behavioral Health: Psychiatry, Child and Adolescent psychiatry
